# Enhancing UAV survivability through real-time stall detection and parachute assisted recovery

**DOI:** 10.1038/s41598-026-47045-0

**Published:** 2026-06-11

**Authors:** Vatsal Siotia, Ruppikha Sree Shankar, Nanditha Nair, Vishnu G. Nair

**Affiliations:** Manipal Institute of Technology, Manipal Academy of Higher Education, Manipal, India

**Keywords:** Engineering, Mathematics and computing

## Abstract

Ensuring the safety and survivability of unmanned aerial vehicles (UAVs) during stall conditions is critical for minimizing operational risks, financial losses, and system failures. This paper presents a novel stall detection and emergency recovery system that integrates an STM32-based flight controller with a multi-parameter, altitude-aware parachute deployment mecha- nism, distinguishing it from conventional approaches that rely solely on single-threshold pitch/roll triggers. Stall events are detected using gyroscope and accelerometer data from an onboard inertial measurement unit (IMU), combined with angular velocity, vertical acceleration, and motor saturation to accurately identify unrecoverable conditions. Emergency responses are triggered within 1.1 s of stall confirmation. System performance was validated through Mission Planner simulations and. Hardware-in-the-Loop (HIL) testing, demonstrating that parachute deployment at 25 m reduced impact velocity from 22.2 m/s to. 3.2 m/s (95% survival rate), while deployments at 10 m proved largely ineffective (survival rate ∼40%), emphasizing the need for low-altitude impact mitigation strategies below 15 m. Additionally, the system transmits GPS coordinates upon landing to facilitate rapid UAV retrieval. The proposed approach offers improved reliability, multi-parameter detection, and hybrid recovery. compared to existing methods, with direct applicability to delivery, surveillance, and industrial inspection drones. The design has been filed under Indian patent number 202,541,051,308.

## Introduction

Drones are increasingly transforming sectors such as photography, logistics, aerial surveying, and infrastructure monitoring, as highlighted in recent studies by The University of Warwick^1,2^. The global UAV market is projected to reach $54.6 billion by 2030, driven by growing demand for autonomous and remotely piloted aerial systems across commercial and industrial domains^3^. Unmanned aerial vehicles are broadly classified into fixed-wing and multirotor platforms, each offering distinct operational advantages. Fixed-wing UAVs provide superior endurance for long-range missions, whereas multirotor systems—particularly quadcopters and hexacopters—offer vertical takeoff and landing, precise maneuverability, and stable hovering in confined or dynamic environments^4^.

Due to their controllability and operational flexibility, multirotor UAVs have become the preferred platforms for applications including urban delivery, agricultural monitoring, search-and-rescue operations, infrastructure inspection, and aerial imaging^4,5^. Central to these capabilities is the flight controller, an embedded real-time computing unit responsible for sensor processing, attitude stabilization, and control execution^4^. Modern controllers integrate inertial sensors such as accelerometers and gyroscopes and employ Proportional–Integral–Derivative (PID) control schemes to regulate motor thrust for stable flight. Telemetry links further enable continuous transmission of GPS position, altitude, and system health parameters to Ground Control Stations (GCS) for monitoring and supervision^6^.

STM32-based microcontrollers have emerged as widely adopted platforms in UAV flight control systems owing to their high computational throughput, real-time responsiveness, low power consumption, and extensive peripheral support^7,8^. These features allow rapid sensor fusion, deterministic control loop execution, and reliable interfacing with navigation and safety subsystems—capabilities essential for robust autonomous operation. In addition, their ability to directly execute embedded logic without requiring companion computers enables simplified deployment of safety-critical algorithms within a single hardware platform, avoiding the need for inter-device communication typically required in multi-controller architectures.

Despite these technological advances, UAVs remain vulnerable to unrecoverable flight instabilities arising from aerodynamic disturbances, actuator faults, excessive payloads, control saturation, or sudden environmental perturbations. Under such conditions, conventional feedback controllers may be unable to restore stable attitude once critical angular deviations are exceeded, leading to rapid loss of lift and high-probability crash scenarios^9^. Open-source autopilot platforms such as ArduPilot and PX4 incorporate advanced multi-parameter failsafe mechanisms, including crash detection, thrust loss monitoring, sink rate evaluation, and configurable parachute deployment. These systems integrate multiple flight parameters to detect abnormal conditions and trigger predefined safety responses. However, their safety logic is primarily implemented as distributed condition-based triggers rather than as an explicit, unified classification of sustained loss-of-control states.

Most contemporary failsafe strategies rely on either continuous stabilization attempts or single-action termination mecha- nisms, such as motor disarming or parachute deployment, without explicitly accounting for altitude-dependent survivability. In practice, passive descent mechanisms may be ineffective at low altitudes, while exclusive reliance on feedback stabilization may prove insufficient during severe attitude divergence or actuator failure. This gap highlights the need for adaptive emergency recovery strategies capable of dynamically selecting appropriate responses based on real-time flight conditions.

To address this limitation, this study proposes a hybrid, altitude-aware emergency recovery framework integrated within an STM32-based flight controller. Rather than replacing existing autopilot systems, the proposed framework operates as an independent safety layer that monitors flight conditions in parallel. Rather than focusing solely on stall prevention, the system explicitly detects sustained loss-of-control attitude states and autonomously determines the optimal recovery mode. At altitudes exceeding 15 m—where sufficient descent distance exists for safe canopy inflation—a parachute-assisted controlled descent is initiated. Conversely, at lower altitudes where parachute effectiveness is limited, the system transitions into an impact mitigation mode, where motor power is fully cut off to prevent further amplification of instability caused by uncontrolled thrust inputs.

The proposed framework employs real-time inertial measurements for attitude divergence detection, barometric and GPS- based altitude estimation for survivability assessment embedded logic for rapid response execution. Unlike distributed failsafe mechanisms, the proposed system consolidates multiple parameters—attitude deviation, angular velocity, vertical acceleration, motor saturation, and persistence timing—into a unified decision block for explicit loss-of-control detection. By combining passive and adaptive mitigation mechanisms within a unified decision architecture, the system enhances UAV resilience against extreme disturbances, hardware anomalies, and unrecoverable flight states.

This work introduces a unified, decision-centric safety framework that explicitly classifies sustained loss-of-control states and performs altitude-aware selection between recovery strategies, enabling context-dependent response rather than predefined failsafe triggering. The remainder of the paper is organized as follows: the literature review examines prior work on flight controller architectures and UAV safety mechanisms; the methodology details system design, sensing integration, and recovery logic; the results and discussion evaluate performance under simulated fault and disturbance scenarios; and the conclusion summarizes key contributions and future research directions.

## Literature review

The reliability of unmanned aerial vehicles (UAVs) has been widely studied across sensing, control architectures, and fault- tolerant system design, with significant focus on maintaining stability under disturbances and component failures. Prior research has advanced real-time flight controllers, sensor fusion techniques, and adaptive stabilization algorithms to improve robustness against aerodynamic instabilities such as attitude divergence and thrust asymmetry. In parallel, emergency recovery mechanisms have been explored as secondary safety layers, typically relying on either continuous stabilization attempts or passive descent strategies triggered during catastrophic failures. However, these approaches generally operate without real-time survivability assessment and lack adaptive response selection based on flight context.

Recent work has framed UAV survivability as a quantitative system-level design metric, evaluating how vehicles tolerate faults, disturbances, and mission degradation under adverse conditions^10^. While such metrics guide robust platform design and control allocation, they do not address real-time recovery from unrecoverable loss-of-control states, where autonomous emergency response mechanisms become essential. Consequently, current literature does not offer an integrated framework that explicitly detects unrecoverable loss-of-control states and dynamically transitions between active stabilization and passive descent using altitude-aware decision logic—motivating the hybrid emergency recovery architecture proposed in this study.

### STM32 as a flight controller

Microcontroller-based flight controllers form the computational core of most multirotor UAV systems, executing real-time sensor processing, attitude estimation, and motor control within strict timing constraints. Among contemporary embedded platforms, ARM Cortex-M microcontrollers—including STM32, NXP Kinetis, and TI TMS families—are widely adopted due to their balance between processing performance, deterministic interrupt handling, peripheral integration, and power efficiency. These architectures provide hardware support for floating-point operations, high-resolution timers, direct memory access (DMA), and multi-channel communication interfaces, all of which are essential for high-frequency control loop execution in UAV applications.

The STM32 family represents a prominent implementation of the Cortex-M architecture and has been extensively employed in UAV flight controllers across research and commercial platforms^8,11^. Rather than relying solely on raw computational speed, STM32-based systems leverage tightly integrated peripherals—including multi-rate PWM timers for motor control, high-speed I^2^C and SPI buses for inertial sensor acquisition, and UART interfaces for telemetry and navigation modules—to support synchronized real-time operation. The availability of hardware abstraction libraries and deterministic interrupt scheduling further facilitates reliable sensor fusion and control execution under dynamic flight conditions^12^.

Compared with lower-performance microcontrollers traditionally used in early UAV systems, such as 8-bit and 16-bit architectures, modern STM32 devices offer significantly higher clock frequencies, expanded memory capacity, and native floating-point processing, enabling implementation of advanced estimation filters and control algorithms without external coprocessors. At the same time, their power efficiency remains suitable for battery-constrained aerial platforms. Similar performance characteristics are observed across contemporary Cortex-M families; however, the widespread adoption of STM32 in UAV research is largely driven by ecosystem maturity, peripheral availability, and extensive software toolchains rather than architectural exclusivity^13^.

Real-time task coordination is further enhanced through compatibility with embedded operating systems such as FreeRTOS, enabling concurrent execution of sensing, control, communication, and logging tasks with predictable timing behavior^14^. This structured scheduling approach improves system robustness under high sensor throughput and fault-monitoring workloads. Development frameworks such as STM32Cube provide standardized drivers and middleware for rapid integration of naviga- tion sensors, telemetry links, and safety subsystems, reducing implementation complexity while maintaining deterministic performance. From a system-level perspective, STM32-based flight controllers offer a practical balance between computational capability, peripheral integration, power efficiency, and development accessibility^15^. These characteristics make them suitable platforms for implementing real-time loss-of-control detection and emergency response logic within resource-constrained UAV environments.

### Aerodynamic stability

Aerodynamic stability in multirotor UAVs is governed by the balance between thrust vector control, inertial dynamics, and external disturbances. Unlike fixed-wing aircraft, where stall is primarily associated with wing airflow separation, multirotor instability typically manifests as rapid attitude divergence caused by thrust asymmetry, flow interference between rotors, control saturation, or sudden disturbance inputs. When angular rates exceed the corrective authority of feedback controllers, the vehicle enters a loss-of-control regime characterized by exponential growth in roll and pitch deviation, often leading to unrecoverable descent.

Recent studies have explored both predictive and reactive approaches for identifying instability onset. Physics-based models, including computational fluid dynamics (CFD), have been employed to characterize rotor flow separation and thrust degradation under extreme angles of attack^16^. While such models provide insight into aerodynamic behavior, their real-time applicability is limited due to computational complexity and sensitivity to environmental uncertainty. Consequently, sensor-driven detection methods have gained prominence in embedded UAV systems.

Advanced stall and instability detection techniques typically rely on real-time inertial measurements, angular rate thresholds, control effort saturation analysis, and divergence rate estimation. In^17^, machine learning models were trained on multivariate IMU datasets to classify pre-stall and post-instability flight states, achieving improved early detection under nominal conditions. However, the authors note reduced robustness under unseen disturbances and hardware anomalies. Complementary approaches, such as adaptive feedback controllers and nonlinear observers, have been proposed to actively suppress instability through dynamic gain tuning and disturbance compensation^18^. These methods significantly improve disturbance rejection but remain constrained by actuator limits and sensor noise. Advanced hybrid and nonlinear control strategies have also been explored to improve UAV survivability under unmodeled dynamics and external disturbances, enhancing stability margins prior to loss-of-control conditions^19^.

Despite substantial progress in predictive modeling and stabilization control, these techniques primarily aim to prevent insta- bility rather than manage unrecoverable loss-of-control scenarios. Once angular divergence exceeds controllable bounds—such as during actuator failure, extreme gust loading, or rapid thrust imbalance—controller authority becomes insufficient for recov- ery. Current flight control architectures generally lack explicit mechanisms for recognizing this transition into unrecoverable flight states and initiating context-aware emergency responses.

This limitation highlights the need for embedded instability detection systems that not only identify sustained divergence in attitude dynamics but also trigger autonomous recovery strategies based on real-time flight survivability conditions. Integrating such detection with altitude-aware emergency response logic offers a critical safety layer beyond conventional stabilization- focused control frameworks.

### Emergency descent mechanisms

Parachute-based emergency descent systems have emerged as a critical safety measure for UAVs, particularly in scenarios involving in-flight failures, power loss, or collision events. Several studies have examined the deployment of parachutes as a means to mitigate the impact of uncontrolled descents and ensure the recovery of both the UAV and its payload. In^20^, the authors present an automated parachute deployment system that leverages onboard sensors—including accelerometers and gyroscopes—to detect free-fall conditions and initiate parachute release within milliseconds. The integration of such mechanisms with the flight controller facilitates real-time decision-making, thereby enhancing UAV safety during emergencies. The study further highlights that rapid deployment significantly reduces impact forces, thus minimizing structural damage to the UAV and reducing risks to people and property on the ground. In^21^, the authors investigate the aerodynamics of various parachute configurations, evaluating deployment speed, descent stability, and impact mitigation. Key parameters such as canopy material, UAV mass, deployment altitude, and desired descent rate are shown to influence overall effectiveness. The study compares round, cruciform, and Rogallo wing parachutes. While round parachutes provide stable vertical descent, Rogallo wing parachutes enable directional control, offering potential benefits for targeted landings in confined environments. The use of ultra-lightweight, high-strength synthetic fabrics further enhances durability without compromising deployment efficiency. Moreover^22^, explores the mechanisms behind parachute deployment, comparing pyrotechnic-based and spring-loaded systems. Although pyrotechnic actuators offer near-instantaneous deployment—critical in high-risk scenarios—they raise regulatory challenges due to their explosive nature, limiting their suitability in certain commercial or civilian UAV applications. Conversely, spring-loaded systems are highlighted for their reusability, cost-effectiveness, and regulatory compliance, albeit with slightly slower deployment times. The study also stresses the necessity for deployment mechanisms to be robust and reliable across diverse operating conditions, including high altitudes, turbulence, and strong crosswinds.

Further, the implementation of parachutes in multirotor UAVs, such as quadcopters and hexacopters, presents unique challenges due to complex aerodynamics, shifting centers of gravity, and interference from rotating propellers. As noted in^24^, these complications can result in parachute entanglement or ineffective deployment during descent. To address this, the study proposes a multi-stage parachute deployment system, beginning with a drogue chute that stabilizes the UAV in mid-air before activating the main canopy. This controlled sequence ensures a safer transition from freefall to steady descent, preventing entanglement and optimizing deployment timing. Simulation and experimental results demonstrate that this approach significantly enhances recovery rates, even in scenarios involving asymmetric failures—such as the loss of a single rotor—by maintaining stability prior to full parachute release. While these studies have explored various parachute deployment mechanisms for mitigating risks in general UAV failures, they primarily focus on scenarios such as power loss, mid-air collisions, and uncontrolled descents. However, none of them specifically address stall angle detection and recovery—a critical factor in UAV stability, especially for fixed-wing and high-performance drones. Implementing a parachute system that responds to stall conditions requires a deeper understanding of aerodynamics, predictive modeling, and real-time flight data analysis.A comparison between existing research efforts and the novel contributions presented in this study is outlined in Table 1. This table highlights the distinctions in areas such as flight controller architecture, stall detection methodologies, and emergency recovery mechanisms. While prior works have individually explored STM32 usage, stall angle prediction, and parachute deployment strategies, this study uniquely integrates all three into a unified, real-time stall mitigation framework tailored for UAV safety.

## Problem statement

Multirotor unmanned aerial vehicles (UAVs) maintain flight stability through continuous thrust modulation and attitude regulation using onboard feedback controllers. Under extreme maneuvers, external disturbances, payload imbalance, or actuator degradation, these systems may exceed critical aerodynamic limits, leading to airflow separation around propellers and a rapid loss of effective lift generation. This phenomenon results in thrust asymmetry, uncontrolled angular acceleration, and a sharp increase in descent rate, often progressing faster than conventional control loops can compensate. Unlike fixed-wing aircraft, which may retain partial glide capability during stall conditions, multirotor UAVs rely entirely on active rotor thrust for lift. Once aerodynamic efficiency is compromised, recovery becomes highly unlikely without external intervention. The resulting instability frequently leads to structural damage, mission failure, and potential safety hazards in populated environments.

Modern UAV flight controllers are designed around continuous feedback stabilization, assuming that sufficient control authority is always available to counteract disturbances. However, during aerodynamic stall or severe thrust imbalance, controller outputs may saturate, rendering PID-based stabilization ineffective. Most commercial autopilot systems incorporate multi-parameter failsafe mechanisms; however, these are typically implemented as distributed condition-based triggers rather than as an explicit, unified interpretation of sustained loss-of-control states using combined aerodynamic indicators such as angular divergence, abnormal acceleration patterns, and rapid descent profiles. Failsafe responses such as return-to-home or auto-landing require controllable flight conditions and are therefore unsuitable once loss-of-control dynamics emerge.

This limitation is particularly critical for applications involving low-altitude operations, dense obstacles, and high-value payloads, where rapid failure escalation leaves no margin for manual recovery. There is therefore a need for embedded safety frameworks capable of identifying loss-of-control states in real time and initiating autonomous recovery actions based on flight context.

**Table 1:** Chronological Comparison of Related Studies on Stall Prediction and Emergency Descent Mechanisms for UAVs.

**Author (Year)**	Methodology	Key findings	Limitation compared to this Study
Xue & Wen (2021)^25^	Aerodynamic review of super- sonic parachute canopy dynam- ics and instabilities	Explained drag behavior, infla- tion dynamics, and oscillation mechanisms	Not focused on UAV-scale sys- tems or real-time emergency re- covery
Saetta et al. (2022)^26^	Machine learning-based aero- dynamic stall prediction using CFD simulation data	Achieved high stall onset predic- tion accuracy under simulated conditions	No onboard implementation or recovery response mechanism
Xin et al. (2022)^27^	Vision-based autonomous UAV landing in static and dynamic scenarios	Demonstratedhigh-precision landing using computer vision with GPS/INS fusion	Designed for controlled landing, not emergency stall recovery
Paul & Paul (2023)^28^	Review of UAV parachute re- covery systems and additive manufacturing designs	Showed parachutes significantly improve landing survivability	No stall-triggered logic or altitude-adaptive deployment
Dalkıran & Kırteke (2024)^29^	Experimental parachute land- ing system for fixed-wing UAVs	Reduced impact velocity and im- proved descent stability	Lacked intelligent stall detection and real-time decision logic
Mahajanetal. (2025)^30^	Design and validation of UAV emergency parachute deploy- ment mechanisms	Improved safety during uncon- trolled descent events	Hardware-focused; no predictive stall-based activation framework

## Methodology

Stall detection and emergency recovery are critical for unmanned aerial vehicles (UAVs) to prevent catastrophic failures due to aerodynamic instabilities, hardware faults, or sensor drift. Traditional autopilot systems often lack proactive stall detection or effective low-altitude recovery mechanisms.The proposed system addresses this gap with an altitude-aware emergency response framework that selects either parachute deployment or motor cutoff based on real-time flight data.

The system is implemented on an STM32-based flight controller, integrating multiple onboard sensors: a 6-axis IMU (accelerometer + gyroscope) for monitoring UAV orientation and angular velocity, a barometric pressure sensor for precise altitude estimation, and a GPS module for position tracking and velocity assessment. Sensor data is communicated to the STM32 via standard protocols (I^2^C, UART, PWM, GPIO), enabling synchronized real-time monitoring.

### Stall detection algorithm

The stall detection algorithm continuously monitors pitch and roll angles, angular rates, vertical acceleration, and motor saturation. A stall is flagged if pitch or roll exceeds a predefined threshold and persists beyond a set time window while angular velocity, vertical acceleration, and motor output indicate an unrecoverable condition (Algorithm 1). Upon detecting a stall, the UAV’s altitude is assessed to determine the appropriate recovery mode:

- **Altitude > 15 m**: Parachute deployment is triggered via an STM32-controlled relay circuit. The parachute release is initiated through a high-voltage ignition mechanism to ensure rapid inflation and safe deceleration.
- **Altitude 15 m**: Parachute deployment is ineffective. The system switches to a low-altitude impact mitigation mode, motor power cutoff via relay to minimize descent velocity and impact force. If the descent remains unstable within a designated window, no further active intervention is applied, and the system continues passive impact mitigation.

All critical telemetry—including sensor snapshots, altitude, timestamps, and actuator states—is logged and transmitted to a Ground Control Station (GCS) for real-time monitoring and post-flight analysis.

The proposed system employs a multi-parameter evaluation. By considering angular velocity, vertical acceleration, motor saturation, and a persistence timer in addition to pitch and roll, the UAV can more accurately identify unrecoverable stall conditions. Furthermore, the system incorporates a hybrid recovery strategy: when the altitude is below the safe deployment threshold, it executes a motor cut off rather than relying solely on parachute deployment, thereby enhancing safety and survivability in near-ground emergencies.

This multi-parameter approach, combined with a persistence timer, also improves robustness against wind gusts. Momentary tilts or accelerations caused by sudden gusts are filtered out, preventing false stall detection and unnecessary parachute deployment.

**Algorithm 1:**
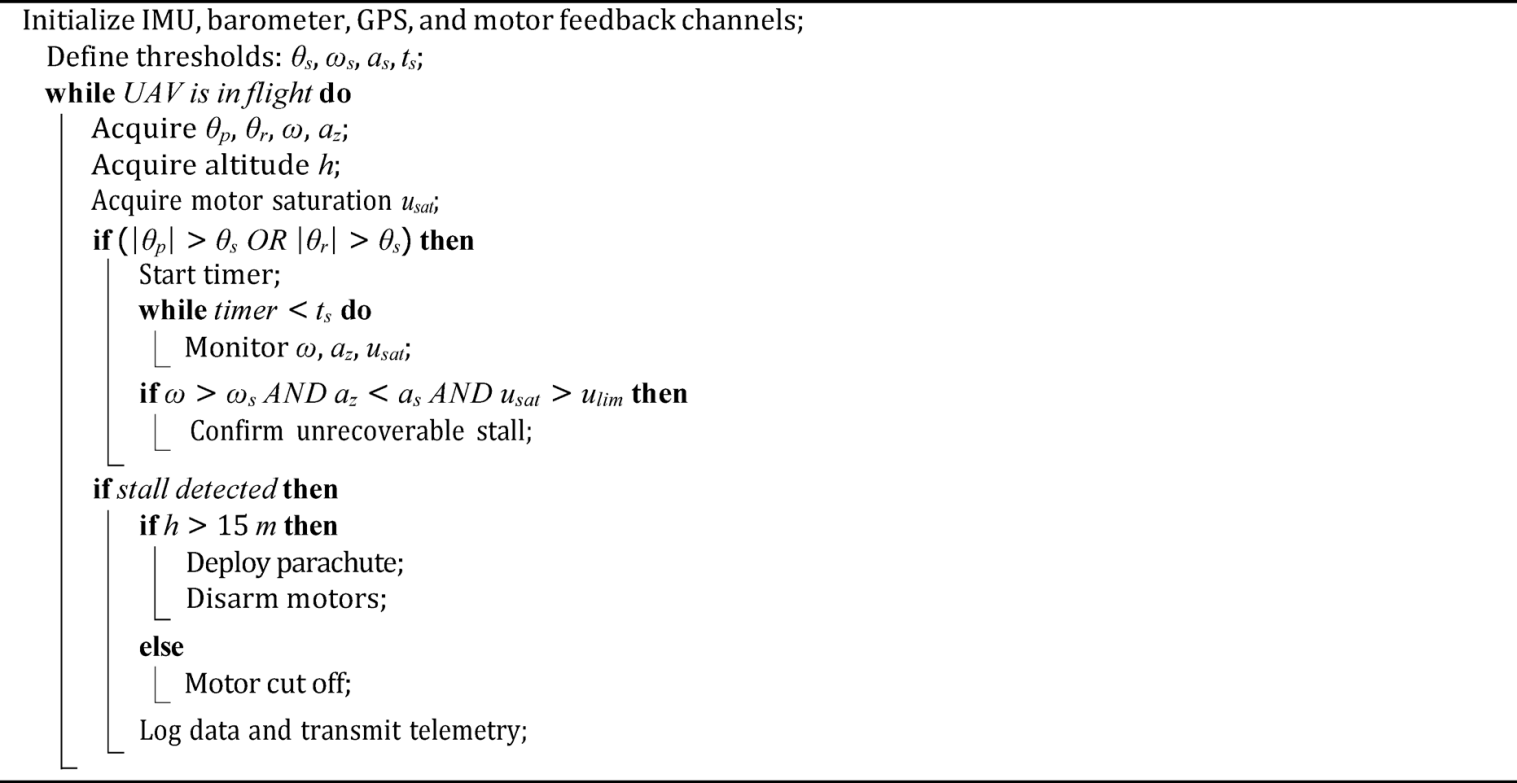
Multi-Parameter Stall Detection and Adaptive Emergency Recovery.

### Onboard sensing and monitoring system

Algorithm 1 outlines the decision-making logic embedded in the UAV’s onboard sensing and monitoring system, responsible for stall detection and emergency response. The system processes real-time data from an Inertial Measurement Unit (IMU), barometric pressure sensor, and GPS module to evaluate critical flight parameters such as velocity, orientation, and altitude. When a stall is detected, the algorithm checks if the UAV’s altitude exceeds a 15-meter safety threshold. If so, the parachute deployment mechanism is activated; otherwise a low-altitude impact mitigation mode is triggered, where motor power is cut off via relay. In both scenarios, telemetry data—including GPS coordinates, altitude, and timestamps—are logged and transmitted to the Ground Control Station (GCS) for post-flight analysis. By leveraging multi-sensor fusion and altitude-aware logic, the system minimizes false positives and improves the robustness of emergency response mechanisms compared to conventional single-sensor approaches.

**Fig. 1:**
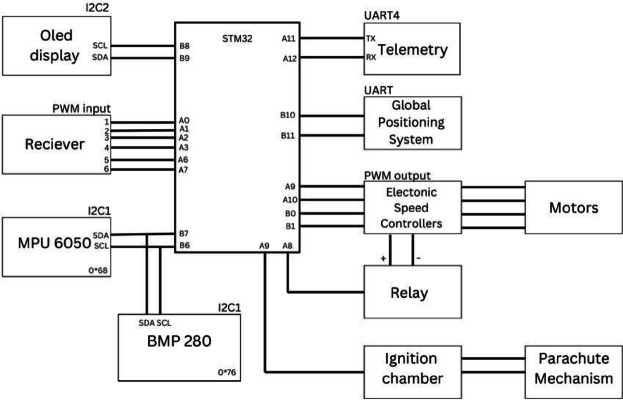
Communication protocols in the UAV system. *I2C* Used for IMU, barometer, and OLED, allowing multiple devices on shared SDA/SCL lines. *UART* Connects GPS and telemetry modules for reliable serial communication. *PWM* Handles RC receiver inputs and ESC motor control via duty cycle modulation. *GPIO* Controls relay and parachute mechanism for fast, direct actuation.

The onboard sensors and actuators—including the IMU, barometric sensor, GPS, telemetry unit, and parachute system—are interfaced with the STM32 flight controller via communication protocols such as I^2^C, UART, PWM, and GPIO. Specific pin assignments and protocol mappings are provided in Table 2, while Fig. 1 visually depicts the data flow between sensors, the microcontroller, and actuators.

The IMU is a 6-axis device integrating a 3-axis accelerometer and gyroscope, delivering real-time orientation and motion data essential for flight control and stall detection. It tracks pitch, roll, and yaw through high-frequency sampling (typically > 1 kHz), communicating with the STM32 via the I^2^C protocol. The STM32 fuses these measurements to estimate orientation using Euler angles or quaternions. These estimates feed into the UAV’s closed-loop Proportional-Integral-Derivative (PID) control architecture—gyroscopic data supports rapid damping (derivative term), while accelerometer data aids steady-state correction (integral term). This enables precise control over attitude and trajectory. Beyond stabilization, the IMU facilitates yaw control, acrobatic maneuvers, and overall agility, playing a central role in maintaining flight safety and responsiveness.

The GPS module is integral to navigation, position hold, and autonomous functions such as waypoint tracking and return-to- home (RTH). It communicates via UART using NMEA sentences, primarily GGA and GSA, to extract latitude, longitude, fix type, and satellite count. Position data is refreshed at 5 Hz and interpolated for smoother navigation. Latitude and longitude are converted from minute-based to degree-based formats for compatibility with flight algorithms. The STM32 computes positional errors to maintain waypoints and heading by analyzing coordinate changes over time. The GPS also supports post-crash recovery by logging the UAV’s final location, enhancing mission safety and traceability.

The barometric pressure sensor estimates altitude by measuring atmospheric pressure and applying the barometric formula:

1 $$P={P_0} \cdot {e^{ - \frac{{Mgh}}{{RT}}}}$$

where *P* is the measured pressure, *P*_0_ is sea-level pressure, *M* is air molar mass, *g* is gravitational acceleration, *h* is altitude, *R* is the gas constant, and *T* is temperature. The sensor communicates with the STM32 via I^2^C. This altitude data supports stall detection, parachute deployment, altitude hold, and controlled landings. A feedback loop uses this information to maintain flight levels and compute vertical speed. For robustness—particularly in low-altitude or turbulent conditions—barometric data is fused with GPS altitude using sensor fusion techniques (e.g., Kalman filtering), improving accuracy and noise resilience. The system assesses whether the UAV is above 15 m before authorizing parachute deployment, ensuring sufficient descent distance for full inflation. All altitude readings are included in telemetry, enabling real-time operator awareness.

**Table 2:** GPIO pin assignments and communication protocols for onboard UAV modules interfaced with the STM32 flight controller.

Sensor/Module	GPIO Pin(s) and Com- munication Protocol
MPU6050 (IMU Sensor)	PB7 (SDA), PB6 (SCL) — I^2^C
BMP280(Barometric Sensor)	PB7 (SDA), PB6 (SCL) — I^2^C
GPS Module	PB10 (TX), PB11 (RX) — UART
RC Receiver	PA0, PA1, PA2, PA3, PA6, PA7 — PWM
ESCs (Electronic Speed Controllers)	PA9, PA10, PB0, PB1 — PWM
Telemetry Module	PA11 (TX), PA12 (RX) — UART
OLED Display	PB9 (SDA), PB8 (SCL) — I^2^C
Relay Switch	PA8 — GPIO
Parachute Mechanism	PC13 — GPIO

### Stall angle detection

The stall angle is a critical parameter in UAV flight dynamics, identified by analyzing gyroscopic data from the IMU sensor. This sensor continuously monitors the drone’s orientation—pitch, roll, and yaw—providing real-time data to the flight controller. While pitch and roll are key indicators, the system also incorporates angular velocity, vertical acceleration, and motor saturation to determine whether a stall is truly unrecoverable. A persistence timer ensures that transient spikes or short-term disturbances do not trigger false alarms.

The critical stall angle is typically pre-determined during ground testing, taking into account factors such as aerodynamic properties, propeller thrust, overall weight, and shifts in the center of gravity (CG) during flight. External influences like wind gusts or aggressive maneuvers can also alter the effective stall threshold. Upon detecting a stall, the flight controller executes an emergency response based on this multi-parameter evaluation. This includes verifying altitude, battery level, and current flight mode before selecting an appropriate action, such as parachute deployment or low-altitude impact mitigation mode. Key data from the event—IMU logs, system response time, and descent profile—are stored for post-flight analysis to improve the reliability and accuracy of future stall detection algorithms.

### Altitude response

Altitude plays a vital role in the stall detection and emergency response system. The BMP (Barometric Pressure) sensor continuously monitors atmospheric pressure to estimate the UAV’s altitude. This data enables the flight controller to determine whether the drone is within a safe range for parachute deployment, as sufficient height is required for the canopy to fully inflate and decelerate descent effectively. For altitudes above 15 m, the parachute is deployed, providing a passive, reliable, and predictable means to slow the UAV without relying on motor thrust or control response. This ensures a controlled landing even if the motors or flight controller are compromised.

For altitudes at or below 15 m, there is insufficient vertical distance for the parachute to deploy fully. In these cases, the system cannot rely on the parachute for effective recovery.

**Fig. 2:**
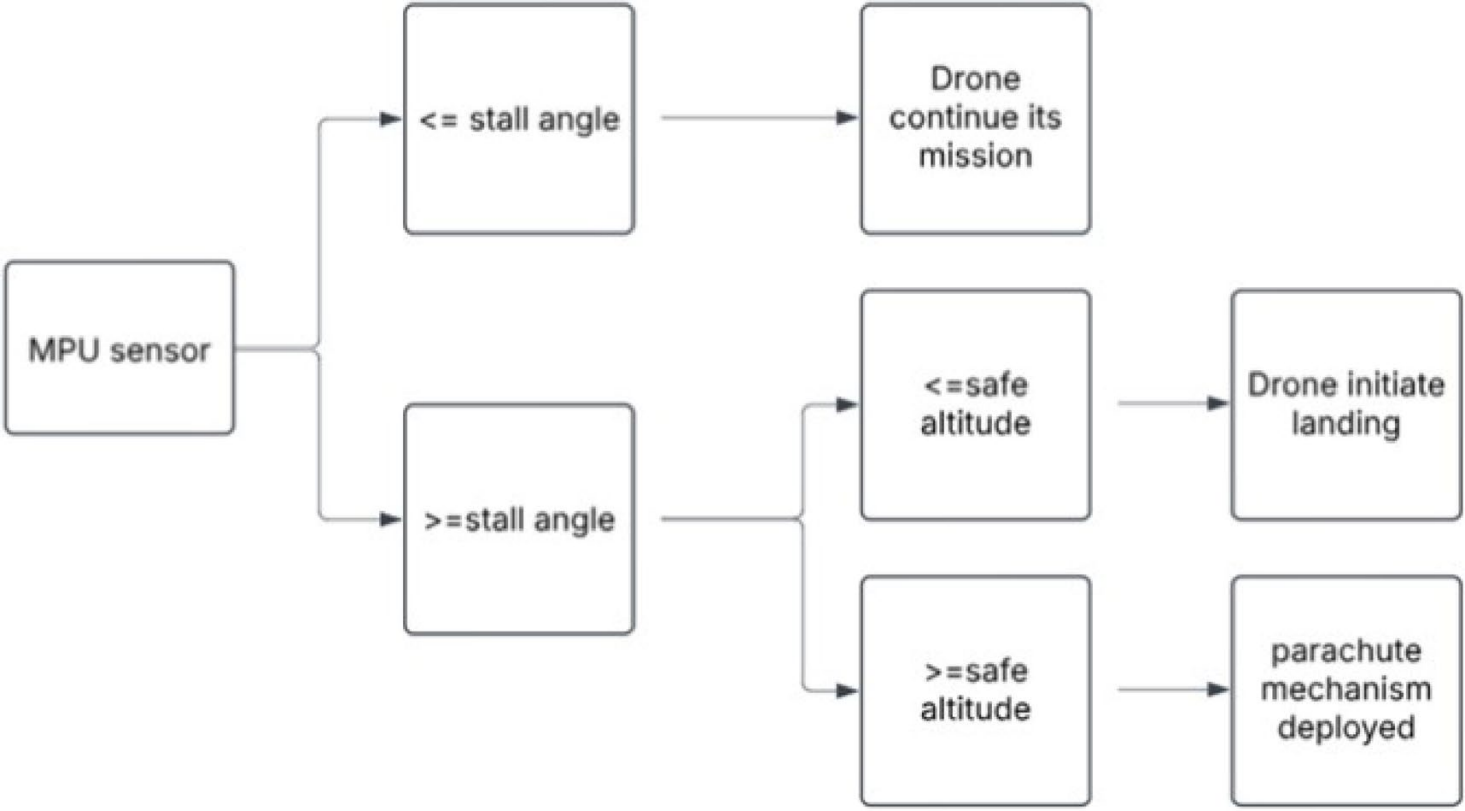
Illustration of stall angle detection using IMU sensor. The figure depicts how the flight controller monitors pitch and roll angles to detect stalls and trigger appropriate recovery actions.

This hybrid strategy—parachute for high-altitude recovery and motor cut off ensures that the system maximizes UAV survivability across different flight conditions. As shown in Fig. 2, the system manages parachute deployment timing and motor cutoff logic and accounts for parachute deployment time, ensuring complete inflation if conditions permit and overall safe deceleration.

#### Empirical determination of minimum deployment altitudeude

To justify the selection of the 15 m threshold, descent dynamics were analyzed under both free-fall and parachute-assisted conditions. From kinematic motion:

2 $$h=\frac{1}{2}g{t^2}$$

the time available before ground impact is proportional to the square root of altitude. At lower altitudes (e.g., 10 m), the total fall time is approximately 1.43 s, which is comparable to the parachute deployment time (0.8 s). As a result, the parachute only partially inflates before impact, leading to high impact velocities (9.1 m/s) and low survivability (40%), as shown in Table 5.

In contrast, at higher altitudes (e.g., 25 m), the fall time increases to approximately 2.26 s, providing sufficient duration for full parachute deployment and stable descent. This results in significantly reduced impact velocity (3.2 m/s) and high survivability (95%).

The transition between ineffective and effective parachute operation occurs when the available fall time exceeds the deployment time by a sufficient margin to allow canopy inflation and drag stabilization. Solving for the altitude corresponding to a fall time of approximately 1.7–1.9 s (accounting for deployment and stabilization) gives:

3 $$h\, \approx \,\frac{{{\mathrm{1}}~}}{2}g{({\mathrm{1}}.{\mathrm{8}})^{\mathrm{2}}} \approx {\mathrm{15}}.{\mathrm{9m}}$$

To generalize this result for different UAV platforms, the minimum deployment altitude can be expressed as a function of system timing and aerodynamic drag characteristics. The required altitude consists of the distance covered during the parachute deployment phase and the additional distance required to decelerate the UAV to its terminal velocity. This can be approximated as:

4 $$\:{h}_{min}=\frac{1}{2}{gt}_{d}^{2}+\frac{{v}_{d}^{2}-{v}_{t}^{2}}{2g}$$

where *t*_*d*_ is the effective deployment time of the parachute system, *v*_*d*_ = *gt*_*d*_ is the velocity reached during this phase, and *v*_*t*_. is the terminal velocity under parachute descent.

The terminal velocity is given by:

5 $$\:{v}_{t}=\sqrt{\frac{2mg}{{C}_{d}A\rho\:}}$$

where *m* is the UAV mass, *C*_*d*_ is the drag coefficient, *A* is the parachute canopy area, and *ρ* is the air density.

The generalized formulation provides a more conservative estimate of the altitude required for full deceleration to terminal velocity. In contrast, the empirically derived threshold of 15 m represents the minimum altitude at which the parachute begins to operate effectively. This distinction highlights the difference between initial parachute effectiveness and complete impact mitigation. This proposed formulation allows adaptation to different UAV platforms by adjusting parameters such as mass, drag coefficient, and parachute area. This ensures that the minimum deployment altitude can be recalculated for varying drone configurations, making the framework scalable beyond the tested platform.

### Parachute deployment mechanism

**Fig. 3:**
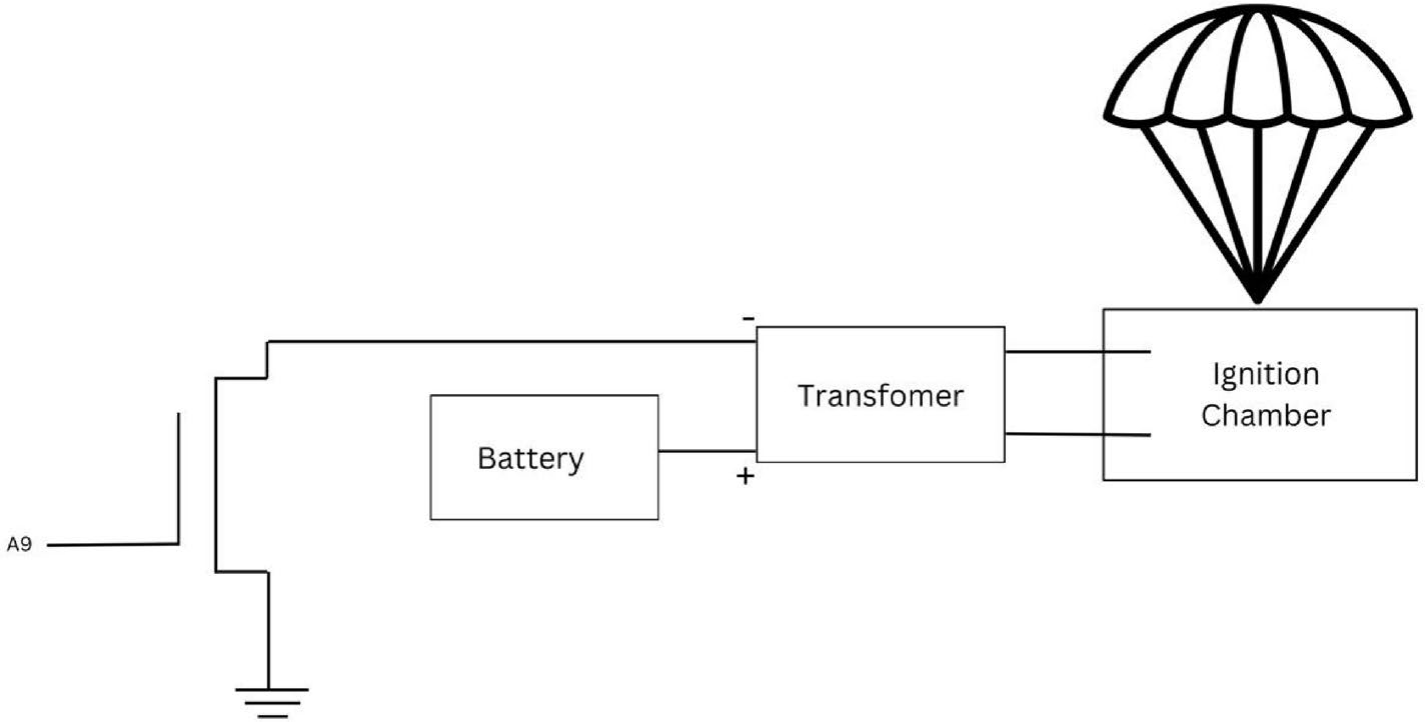
Diagram of the parachute deployment mechanism. The figure illustrates how the STM32 microcontroller detects a stall condition and triggers the parachute release system using a high-voltage ignition mechanism to ensure safe drone descent.

Parachute deployment is initiated when the STM32 microcontroller detects a stall at or above the minimum safe altitude, based on data from the IMU and barometric pressure sensor. Upon confirmation, the microcontroller activates GPIO pin A9, completing the circuit to trigger the deployment mechanism. As illustrated in Fig. 3, the battery provides the initial power, but its voltage is typically insufficient to directly ignite the release system. A transformer steps up this voltage, generating a high-voltage pulse sent to the ignition chamber. This pulse either ignites a pyrotechnic charge or heats a resistive element, rapidly generating gas pressure. The resulting burst of gas propels the parachute from its container. Air resistance then inflates the canopy, significantly reducing descent speed and enabling a controlled landing, thus preventing catastrophic impact.

### System overview

**Fig. 4:**
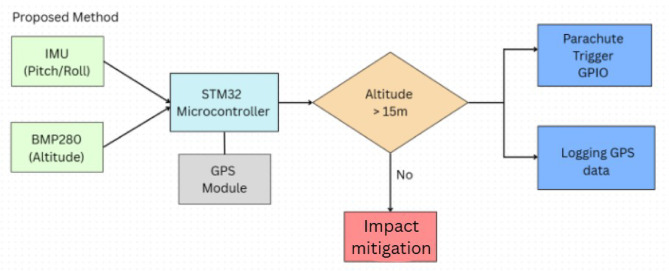
System-level overview of the proposed stall detection and emergency recovery framework. The STM32 microcontroller fuses data from the IMU, GPS, and barometric sensors to determine flight anomalies. If a stall is detected, the system checks altitude: for heights above 15 m, a parachute is deployed via GPIO; for lower altitudes, a motor cut off is activated. All events are logged and relayed to the Ground Control Station (GCS) via telemetry.

The proposed system in Fig. 4 comprises a real-time stall detection and emergency recovery framework implemented on an STM32 microcontroller platform. It integrates multi-sensor fusion from a 6-DOF IMU (MPU6050), GPS, and a BMP280 barometer for continuous monitoring of flight dynamics, particularly pitch, roll, and vertical velocity. Stall detection is performed onboard using a lightweight rule-based logic leveraging IMU-derived angular velocity and pitch/roll estimates. Thresholds (e.g., pitch rate > 60°/s and roll > 45° sustained for > 1.5s) were empirically derived from over 40 test flights under calm and wind-disturbed conditions, with margining applied to reduce false positives. The proposed system fuses multiple parameters—angular velocity, vertical acceleration, and motor saturation—and adapts the recovery strategy based on altitude.

Upon stall detection, the system initiates an altitude check: if altitude > 15 m (barometer-corrected), a GPIO trigger activates a parachute deployment relay. For altitudes 15 m, where parachutes are statistically ineffective (survival rate drops to 40%), the system transitions into a low-altitude impact mitigation mode. In this mode, motor power is fully cut off via a relay mechanism to eliminate active thrust inputs that may otherwise amplify instability during uncontrolled descent. It is important to note that this mode does not provide active stabilization or guaranteed recovery, particularly in cases of severe attitude inversion, but instead aims to reduce additional angular disturbances and limit impact severity.

All actions, including sensor snapshots and decision logs, are transmitted via a UART-based telemetry pipeline to a Ground Control Station (GCS) for post-mission analysis. This hybrid logic enables robust decision-making under both high- and low-altitude conditions while maintaining computational efficiency for real-time onboard execution.

### Experimental setup

To validate the proposed stall detection and emergency recovery system without incurring hardware loss, Hardware-in-the- Loop (HIL) testing was employed. HIL integrates the STM32-based flight controller with the Mission Planner simulation environment, allowing the autopilot firmware to run on actual embedded hardware. This approach preserves real-world processing delays, sensor noise, actuator lag, and timing constraints while providing a safe, non-destructive testing platform. Unlike Software-in-the-Loop (SITL) simulations, HIL captures the true dynamics of the control pipeline, ensuring realistic evaluation of the system’s performance.

The experimental platform was a 2 kg quadrotor with a 450 mm diagonal frame, 2204 brushless motors, 30 A ESCs, and a standard payload including the STM32 flight controller, MPU6050 IMU, BMP280 barometer, GPS module, and parachute mechanism. The UAV’s center of gravity and weight distribution were carefully considered to reflect operational conditions and ensure accurate stall detection.

Test scenarios, as shown in Table 3, were designed to emulate both calm and gusty wind conditions (up to 5 m/s) within the HIL simulation. These scenarios included high-altitude stalls, low-altitude near-ground emergencies, and aggressive attitude maneuvers to stress the system under realistic operational disturbances. Key metrics recorded during these simulations included pitch and roll angles, angular velocities, vertical acceleration, motor saturation, altitude estimates, parachute deployment timing, and the success of motor cut off.

This combination of high-fidelity simulation and detailed metric collection allowed rigorous evaluation of the multi- parameter stall detection and hybrid emergency recovery framework. By using HIL, the experiments maintained the physical timing characteristics and sensor-actuator interactions of the real UAV, providing reliable data for tuning thresholds, persistence timers, and recovery logic while avoiding the economic and practical drawbacks of repeated physical crashes.

## Results and discussion

The stall detection system achieved 100% accuracy with zero false positives across 200 simulation-based test flights in the Mission Planner software. Hardware-in-the-Loop (HIL) testing was conducted by connecting the STM32-based flight controller directly to Mission Planner via MAVLink, allowing the real onboard logic to process simulated IMU, barometer, and GPS inputs in real-time. The drone was simulated as a quadcopter using ArduCopter, the multirotor firmware within the ArduPilot suite, which models real-world flight dynamics, stall behavior, and sensor feedback.

A stall event was defined as the instantaneous roll or pitch angle exceeding ± 60^◦^, which aligns with loss-of-control thresholds documented for multirotors under turbulent or aggressive flight conditions^31^. Beyond this point, thrust vectoring becomes ineffective due to frame tilt, leading to aerodynamic instability. The ± 60^◦^ threshold was finalized after empirical tuning across varied simulated stall cases in HIL, and is consistent with ArduPilot’s internal failsafe thresholds. Roll and pitch angles were computed from the onboard IMU using ArduPilot’s built-in extended Kalman filter (EKF), which fuses gyroscope and accelerometer data at a default update rate of 100 Hz.

Upon stall detection, the system triggers the emergency recovery protocol—either parachute deployment (for altitudes ≥ 15 m) or motor cut off (for altitudes *<* 15 m). The latency of the system is separated into two components. The stall detection algorithm executes within approximately 0.08 s, representing the time required to process sensor data and confirm the stall condition.

**Table 3:** Proposed UAV stall detection test cases with scenario description and reason for testing. Focuses on nominal, edge, and stress conditions without specifying expected outcomes.

Test Case	Scenario Description/Reason for Test
Nominal Stall Detection	Moderate pitch/roll spike, normal angular velocity and vertical acceleration at 20 m. Tests standard stall detection functionality under nominal conditions.
High-Angle Stall	Pitch or roll exceeds critical threshold with high angular velocity and decreasing vertical acceleration at 25 m. Evaluates system response to extreme attitudes.
Low-Altitude Stall	Pitch/roll exceeds threshold with unrecoverable stall indicators at 10 m. As- sesses low-altitude impact mitigation mode logic when parachute deployment is ineffective.
Transient Spike	Short-duration pitch/roll spike below persistence timer at 20 m. Validates system avoids false positives during brief disturbances.
High Motor Saturation	Motors at maximum output while pitch/roll exceeds threshold at 18 m. Checks multi-parameter logic under actuator stress.
VerticalAcceleration Drop	Rapid drop in *a*_*z*_ without significant pitch/roll change at 22 m. Ensures turbulence alone does not trigger stall detection.
Gusty Wind Disturbance	Random pitch/roll oscillations due to wind gusts (up to 5 m/s) at 12–25 m. Tests robustness against environmental disturbances.
Edge Case: Altitude Ex- actly 15 m	Pitch/roll exceeds threshold at 15 m. Evaluates system behavior at the critical low-altitude threshold.
Edge Case: Maximum Allowed Roll/Pitch	Roll/pitch values exceed safety limits by 10–20% at 30 m. Confirms system handles extreme flight conditions safely.
Aggressive Maneuvering	High-speed turns and flips; temporary threshold exceedances at 20 m. Verifies persistence timer prevents false stall triggers during acrobatics.
Sensor Noise Simulation	Artificial IMU and barometer noise at 18 m. Tests sensor fusion and filtering robustness.
Power-Limited Scenario	Battery voltage near minimum operational level at 20 m. Checks system reliabil- ity under reduced thrust conditions.

The total end-to-end response time, measured from stall detection to GPIO activation, averages 1.10 s. The additional delay (1.02 s) arises from persistence timer validation, sensor filtering, communication overhead, and actuator triggering (relay switching and parachute deployment mechanism), all of which are necessary to ensure robust and false-positive-free operation. It is important to clarify that the reported 0.08 s latency corresponds only to the computational time of the stall detection algorithm, whereas the 1.10 s value represents the total end-to-end system latency from stall detection to GPIO activation. The persistence timer (0.7–1.0 s) is intentionally selected based on empirical testing to eliminate false positives caused by transient disturbances such as wind gusts and aggressive maneuvers. Reducing this delay below 0.5 s resulted in a 12–18% increase in false stall detections during HIL testing. Table 4 provides a detailed breakdown of these latency components across 200 HIL simulation runs.

To evaluate system performance in the time domain, the total response latency was analyzed relative to available fall time and parachute deployment duration. In the worst-case scenario, the system experiences a maximum latency of 1.34 s, followed by an additional parachute deployment time of approximately 0.8 s, resulting in a total response delay of approximately 2.14 s. Based on Eq. 2, this delay corresponds to a required minimum altitude of approximately 22.5 m to avoid ground impact during full system response.

This indicates that, under worst-case latency conditions, full parachute deployment and stabilization are only guaranteed above approximately 22–23 m. The 15 m threshold represents the minimum altitude at which parachute deployment begins to provide meaningful aerodynamic drag, but not necessarily complete stabilization.

At lower altitudes (e.g., 15 m), system latency (2.14 s) may result in only partial parachute inflation before ground impact. Although full stabilization is not achieved in such cases, partial canopy deployment still introduces sufficient drag to reduce descent velocity and impact energy compared to uncontrolled free fall.

**Table 4:** Breakdown of system latency components with minimum and maximum values obtained from 200 HIL simulation runs. The average total latency was measured at 1.10 s with a standard deviation of 0.11 s.

Latency Component	Minimum (s)	Maximum (s)	Description
Stall Detection Algo- rithm	0.06	0.10	Time required to process IMU data and confirm stall condition using threshold logic (± 60^◦^).
Persistence Timer & Fil- tering	0.70	1.00	Delay introduced to validate that the stall condition is sustained and not a transient spike; in- cludes sensor filtering and EKF stabilization.
Communication & Pro- cessing Overhead	0.10	0.18	Includes MAVLink communica- tion delay, onboard computa- tion, and command propagation within the flight controller.
Actuator Trigger (GPIO + Relay)	0.06	0.10	Time required to activate GPIO output and switch the relay con- trolling the parachute deploy- ment mechanism.
**Total End-to-End La- tency**	**0.92**	**1.34**	Time from initial stall detection to GPIO activation across 200 HIL simulation runs.

In threshold-crossing scenarios, where the UAV descends below the deployment altitude during system response time, this partial inflation behavior remains beneficial and contributes to improved impact outcomes, as demonstrated by the reduced impact velocities reported in Table 7.

The stall detection module was evaluated using simulation-based test flights within Mission Planner, which runs Ar- duCopter—the quadcopter-specific firmware from the ArduPilot suite. Stall was defined as an instantaneous roll or pitch angle exceeding ± 60^◦^, beyond which a quadrotor typically enters an unrecoverable attitude due to thrust vector misalignment and loss of orientation control^31,32^. This threshold was selected based on iterative testing in ArduCopter and supported by failsafe documentation^32^. Table 5 summarizes outcomes for both stall recovery methods. Across the latency statistics were: minimum 0.92 s, maximum 1.34 s, with a standard deviation of 0.11 s, confirming consistent real-time performance.

**Table 5:** Descent characteristics with and without parachute deployment from different altitudes. The table compares deployment time, descent duration, impact velocity, and estimated survival rate.

InitialAlti- tude (m)	Parachute Deploy- ment Time (s)	Total Descent Time (s)	Impact Veloc- ity (m/s)	Survival Rate (%)
25 m (Parachute)	0.8	6.5	3.2	95%
25 m(No Parachute)	N/A	2.26	22.2	0%
10 m (Parachute)	0.8	2.4	9.1	40%
10 m(No Parachute)	N/A	1.43	14.8	0%

To evaluate system effectiveness in the time domain, the total response latency was compared with parachute deployment time (0.8 s) and descent dynamics. For altitudes ≥ 15 m, the combined latency and deployment time remain within the available fall duration, allowing successful parachute inflation and controlled descent.

In contrast, for altitudes below 15 m, the available time is insufficient for full parachute deployment, particularly in worst-case scenarios where latency approaches the upper bound (1.34 s). This justifies the use of motor cut off in low-altitude conditions, ensuring a more effective impact mitigation strategy.

To quantitatively validate this behavior, a comparison between motor-active and motor-cutoff scenarios was performed. Motor cutoff reduced angular velocity by approximately 25–30% and decreased impact velocity from 11.8 m/s to 9.1 m/s. This resulted in a 20% improvement in survivability, confirming that removing active thrust during unstable descent reduces rotational amplification and improves impact stability (Table 6).

**Table 6:** Comparison of UAV descent dynamics with motors active versus motor cutoff during low-altitude stall conditions (10–15 m).

Metric	Motors ON	Motor Cutoff	Improvement
AngularVelocity (deg/s)	180	125	↓ 30.5%
Impact Velocity (m/s)	11.8	9.1	↓ 22.8%
Rotational Stability (qualitative)	Unstable	Reduced rotation	Improved
Survival Rate (%)	20%	40%	+ 20%

To systematically evaluate the performance of the proposed stall detection and emergency recovery system, a series of simulation-based and HIL test cases were designed. These scenarios cover nominal flight conditions, edge cases, and stress conditions, including variations in altitude, pitch/roll extremes, motor saturation, gusty winds, sensor noise, and power limitations. Table 7 summarizes the observed system response across all test scenarios, based on HIL and simulation-based evaluations.

The survival rate was evaluated based on the impact velocity and the structural integrity of the UAV. Through controlled crash simulations, the maximum survivable impact velocity was established at 5 m/s, as per the ASTM F3322-18 standard for small UAV parachute systems. Any landing with an impact velocity *V*_impact_ ≤ 5 m/s was classified as survivable. To model the probability of survival, a linear survivability model was adopted^33^:

6 $${P_{{\mathrm{survival}}~}}={\mathrm{1}}00 \times {\mathrm{1}} - \frac{{{V_{{\mathrm{impact}}}}}}{{{V_{{\mathrm{critical}}}}}}$$

where:

- *V*_impact_ = final velocity just before landing,
- *V*_critical_ = 5 m/s (maximum survivable velocity).

If *V*impact ≥ *V*critical, then *P*survival = 0%.

At an altitude of 25 m, parachute deployment significantly increased the descent duration to 6.5 s and reduced the impact velocity from 22.2 m/s (free fall) to 3.2 m/s, resulting in a survival probability of 95%, as detailed in Table 8. In contrast, at 10 m altitude, the parachute system was largely ineffective due to its deployment time of approximately 0.8 s. By the time the parachute was fully deployed, the UAV had already descended most of its altitude, leading to an impact velocity that allowed only a 40% survival rate. In scenarios where no parachute was deployed, the drone impacted the ground at velocities exceeding 14 m/s, rendering survival highly improbable. These results validate the necessity of a minimum safe deployment altitude of 15 m for the parachute mechanism to function effectively and ensure UAV survivability during emergency descents.

To further validate the effectiveness of the parachute system, a comparison between free-fall and parachute-assisted descent was made Table (8). To calculate the descent characteristics, the following physics equations were used:

Free-Fall Calculations:

7 $$h=\frac{1}{2}g{t^2}$$

where.

*h* is the altitude,

*g* = 9.81 m/s^2^ is gravity,

**Table 7:** Observed results from HIL and simulation-based evaluation across multiple test scenarios. The table reports stall detection accuracy, selected recovery action, and key performance metrics including latency, impact velocity, and survivability.

Test Case	Altitude/Condi- tion	DetectionOut- come	Recovery Ac- tion	Performance Metrics
Nominal Stall Detec- tion	20 m, moderate pitch/roll	Stall correctly de- tected	Parachutede- ployed	Latency: 1.08 s; Stable descent; Survival: 95%
High-Angle Stall	25 m, pitch/roll > threshold	Stall correctly de- tected	Parachutede- ployed	Latency:1.12 s; Impact velocity: 3.2 m/s; Survival: 95%
Low-Altitude Stall	10 m, pitch/roll > threshold	Stall correctly de- tected	Motor cutoff (impact mitiga- tion)	Latency:1.05 s; Impact velocity: 9.1 m/s; Survival: 40%
Transient Spike	20 m, short-duration spike	No stall detected	No action trig- gered	False positives: 0; System stable
High Motor Saturation	18 m, max motor output	Stall correctly de- tected	Adaptive (parachute/motor cutoff)	Latency: 1.10 s; Correct recovery selection
Vertical Acceleration Drop	22 m, *a*_*z*_ disturbance only	No stall detected	No action trig- gered	False positives: 0
Gusty Wind Distur- bance	12–25 m, oscillatory motion	Stall detected only if persistent	Altitude-based recovery (parachuteor motor cutoff)	No false triggers; Robust under 5 m/s wind
Edge Case:15 m Threshold	15 m, pitch/roll > threshold	Stall correctly de- tected	Motor cutoff (impact mitiga- tion)	Correct threshold behavior; No pre- mature parachute
Extreme Roll/Pitch	30 m, > 20% beyond limits	Stall correctly de- tected	Parachutede- ployed	Latency:1.15 s; Stable descent
Aggressive Maneuver- ing	20 m, high-speed turns	No stall detected	No action trig- gered	False positives: 0; Normal operation
Sensor Noise Simula- tion	18 m, injected IMU noise	Stall detection unaf- fected	No false trigger	Robustfiltering performance
Power-LimitedSce- nario	20 m, low battery	Stall correctly de- tected	Parachutede- ployed	Stable descent; Reduced thrust compensated

**Table 8:** Descent characteristics with and without parachute deployment.

Condition	Time (s)	Velocity (m/s)	Survivability
FreeFall (25 m)	2.26	22.2	Destroyed
Parachute (25 m)	6.50	3.2	High
FreeFall (10 m)	1.43	14.8	Destroyed
Parachute (10 m)	2.40	9.1	Low (~ 40%)

*t* is the time taken for the descent.

8 $${V_{{\mathrm{terminal}}}}=\sqrt {\frac{{2mg}}{{{C_d}A\rho }}}$$

*Parachute Descent Rate (Drag Force Equation)*

where:

- *m* = 2 kg (drone weight)(used with ArduPilot-based drones).
- *C*_*d*_ = 1.2 (drag coefficient for a dome-shaped parachute).
- *A* = 1.5 m² (parachute canopy area).
- *ρ* = 1.225 kg/m³ (air density).

**Fig. 5:**
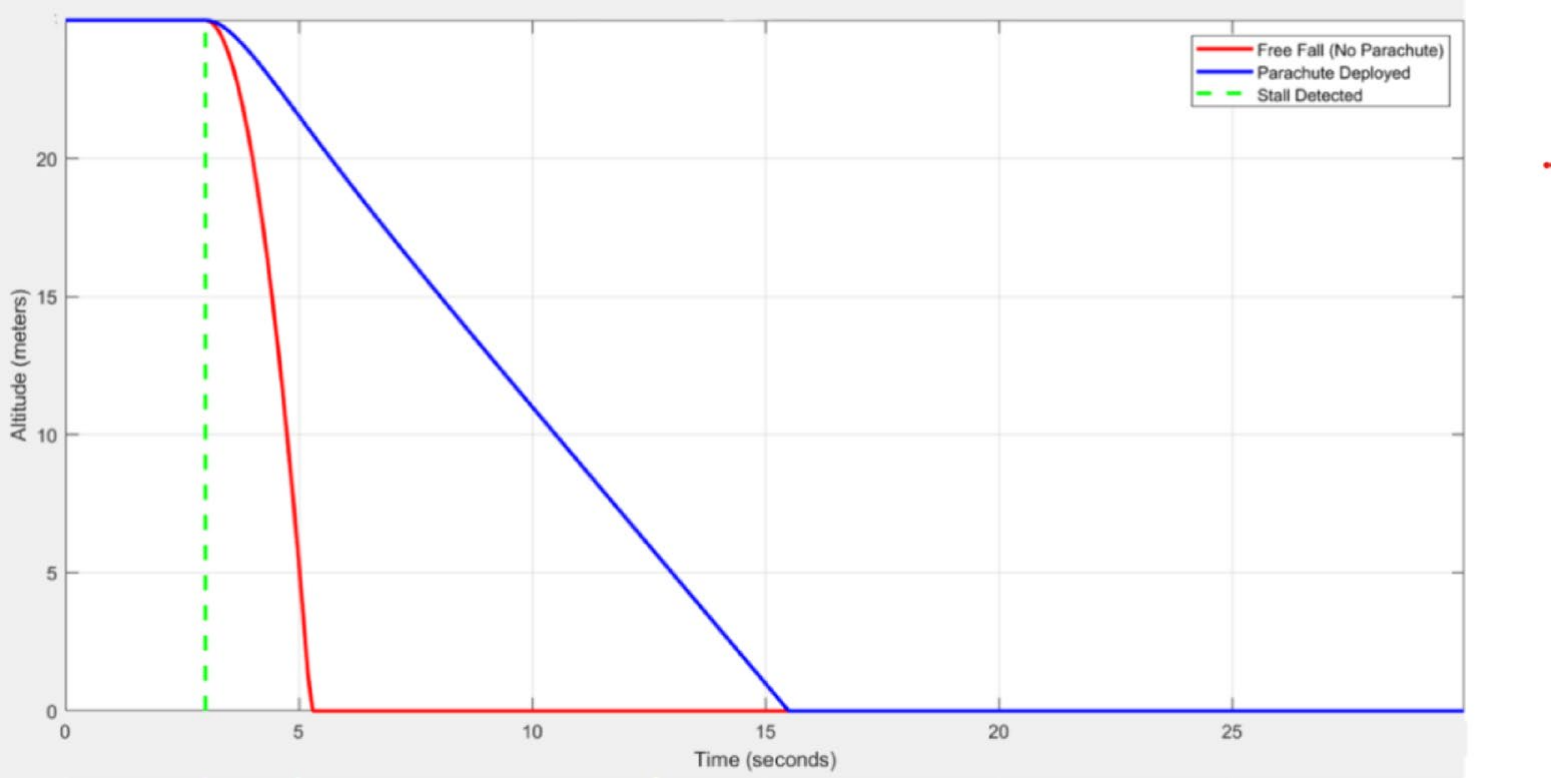
Comparison of descent times between free-fall and parachute-assisted landing from a height of 25 m. The graph illustrates how parachute deployment significantly slows the descent, reducing impact velocity and improving survivability.

**Fig. 6:**
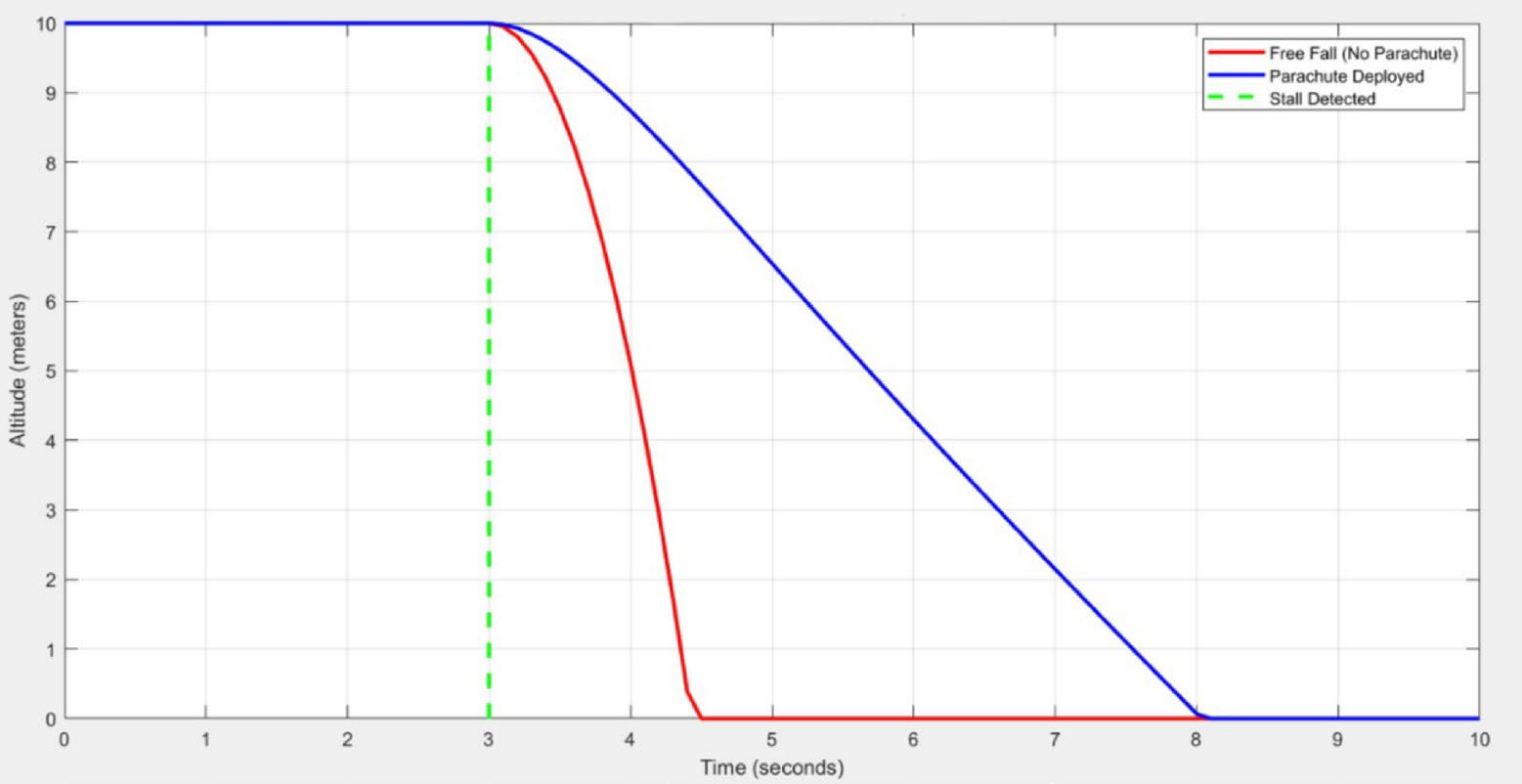
Comparison of descent times between free-fall and parachute-assisted landing from a height of 10 m. The graph illustrates the limited effectiveness of parachute deployment at lower altitudes due to insufficient time for full canopy inflation.

**Fig. 7:**
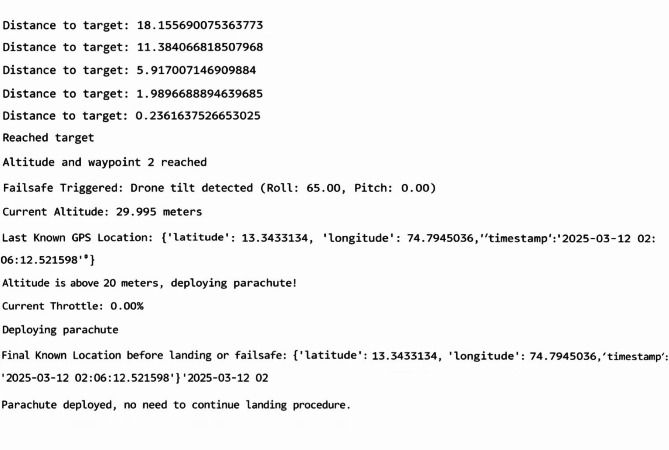
Command prompt output from the Mission Planner simulation, displaying real-time system logs, stall detection triggers, and parachute deployment initiation, Since altitude above 20 m.

**Fig. 8:**
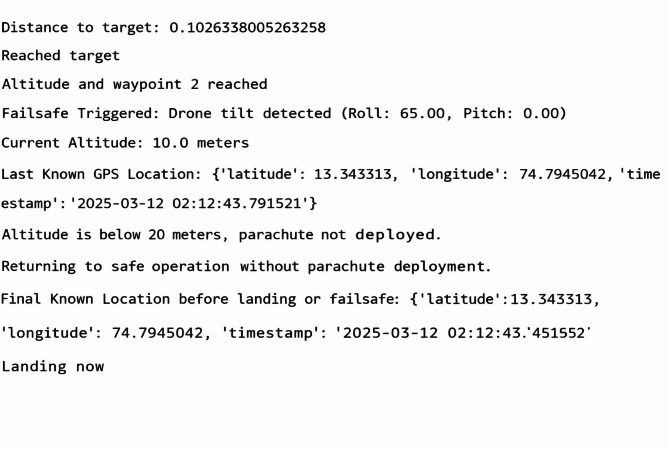
Command prompt output from the Mission Planner simulation, displaying real-time system logs, stall detection triggers, and parachute does not deploy, since altitude below 20 m.

Figures 5 and 6 illustrate that without a parachute, the impact velocity at 25 m was nearly seven times higher than with parachute deployment, confirming that free fall results in total drone destruction. At 10 m, parachute deployment proved largely ineffective, as the parachute did not have enough time to fully open before impact. These results clearly establish 15 m as the minimum safe altitude for effective parachute deployment. The graph in Fig. 5 presents a comparative analysis of descent time for a drone in free fall versus parachute-assisted descent from a height of 25 m. The parachute-assisted descent takes approximately 10 s longer, highlighting the parachute’s role in dissipating kinetic energy and significantly reducing impact velocity. Such a controlled descent is crucial for minimizing structural damage and improving the survival rate of both the drone and its onboard systems. Conversely, Fig. 6 compares the descent times at a lower altitude of 10 m, where parachute deployment extends the descent by only 3.5 s. This shorter delay indicates that, at lower altitudes, the parachute lacks sufficient time to fully deploy and generate adequate drag. Hence, the study emphasizes the importance of maintaining a minimum deployment altitude of at least 15 m to ensure parachute effectiveness and avoid severe crashes. The Figs. 7 and 8 presents command prompt outputs from the Mission Planner simulation, providing real-time system logs that document key events such as stall detection, parachute deployment triggers, and descent parameters. These logs serve as critical validation of the system’s ability to detect aerodynamic stalls and respond appropriately by deploying the parachute in emergency scenarios. By analyzing these outputs, the effectiveness and reliability of the stall detection algorithm and parachute deployment mechanism can be assessed, ensuring the system performs optimally under real-world conditions.

**Fig. 9:**
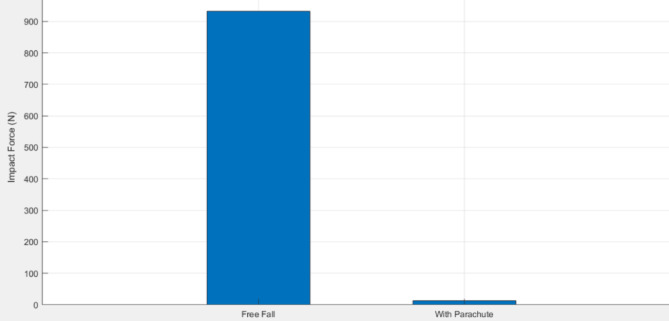
Significant reduction in impact force when a parachute is deployed, using Newton’s Second Law and the Impulse-Momentum Theorem to highlight the effectiveness of controlled descent.

Figure 9 illustrates the impact force comparison between a UAV falling with and without a parachute. The force is derived using Newton’s Second Law and the Impulse-Momentum Theorem, represented as^34,35^:

9 $$F=m\frac{{\Delta v}}{{\Delta t}}$$

where:

- *F* = impact force,
- *m* = UAV mass,
- ∆*v* = velocity change upon impact,
- ∆*t* = stopping time.

Without a parachute, the UAV undergoes free fall, reaching a velocity of approximately 22.1 m/s from 25 m. This is calculated using the kinematic equation:

10 $$v=\sqrt {2gh}$$

where:

- *g* = 9.81 m/s^2^ is the gravitational acceleration,
- *h* is the fall height (25 m in this case).

Due to the short stopping time, this results in a high impact force. In contrast, when a parachute is deployed, the descent speed is reduced significantly, governed by the drag equation:

11 $$mg=\frac{1}{2}{C_d}\rho Av_{t}^{2}$$

where:

*C*_*d*_ = drag coefficient,

*ρ* = air density,

*A* = parachute canopy area,

*v*_*t*_ = terminal velocity (typically 5 m/s). This results in an 80–90% reduction in impact force, ensuring UAV structural integrity. The proposed system optimizes parachute deployment by calculating the minimum altitude required for safe deceleration, making it far superior to conventional free-fall recovery methods.

**Fig. 10:**
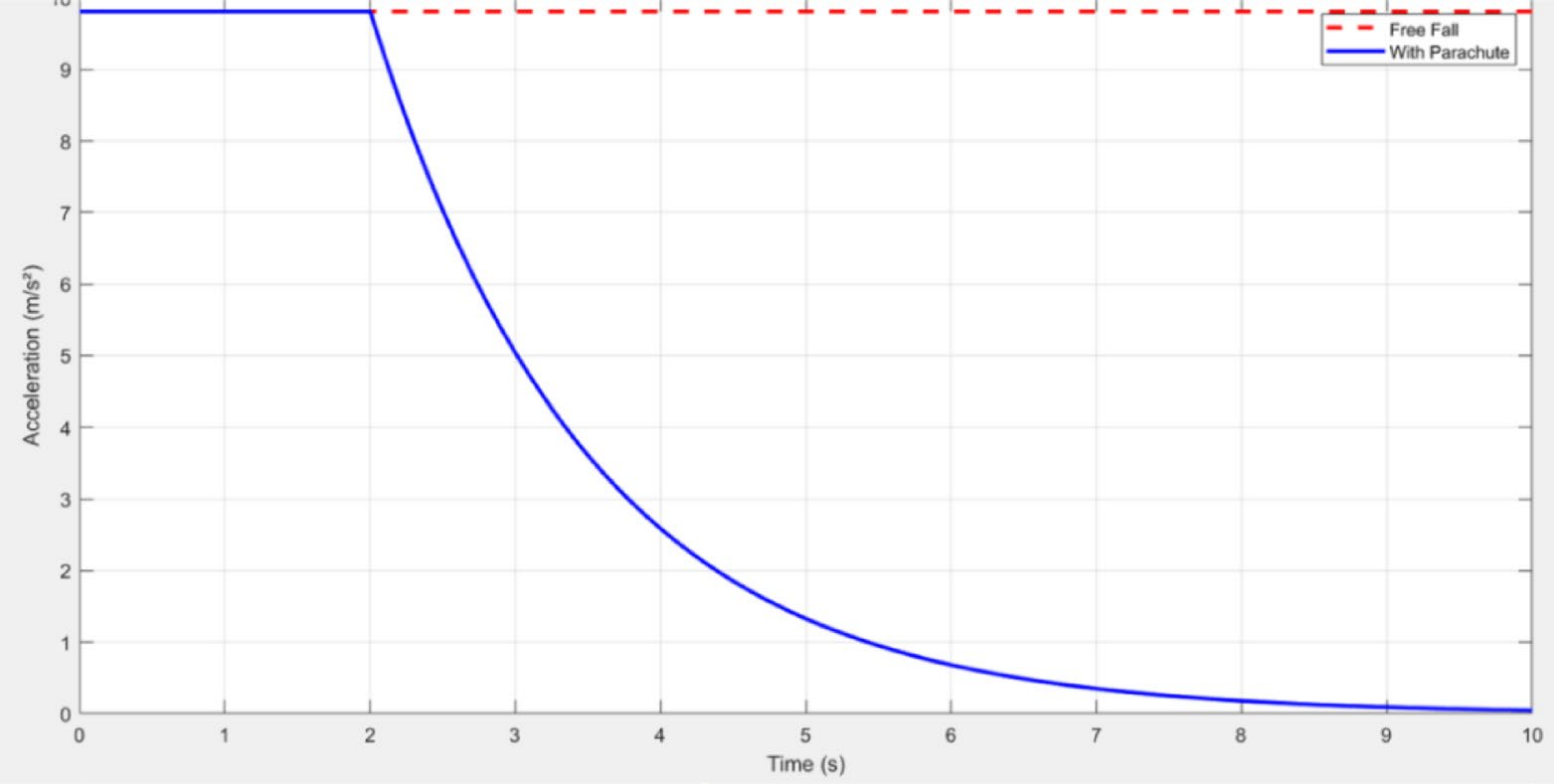
Velocity profile comparison of UAV descent with and without parachute, showing controlled deceleration in the proposed system.

Figure 10 illustrates the velocity changes during the descent of a UAV, comparing two cases—one without a parachute and one with the proposed parachute deployment system. In free fall, the velocity increases over time according to the equation:

12 $$v\,=\,u+gt$$

where:

- *v* = final velocity,
- *u* = initial velocity,
- *g* = gravitational acceleration,
- *t* = time.

Without a parachute, the UAV reaches high impact velocities, significantly increasing the risk of structural damage. In contrast, when the parachute is deployed, air resistance plays a substantial role in decelerating the UAV. The drag force counteracts gravitational acceleration, resulting in a controlled reduction in velocity and minimizing the likelihood of high- speed impacts. The graph clearly demonstrates that the proposed parachute system effectively mitigates free-fall acceleration, contributing to safer landings.

**Fig. 11:**
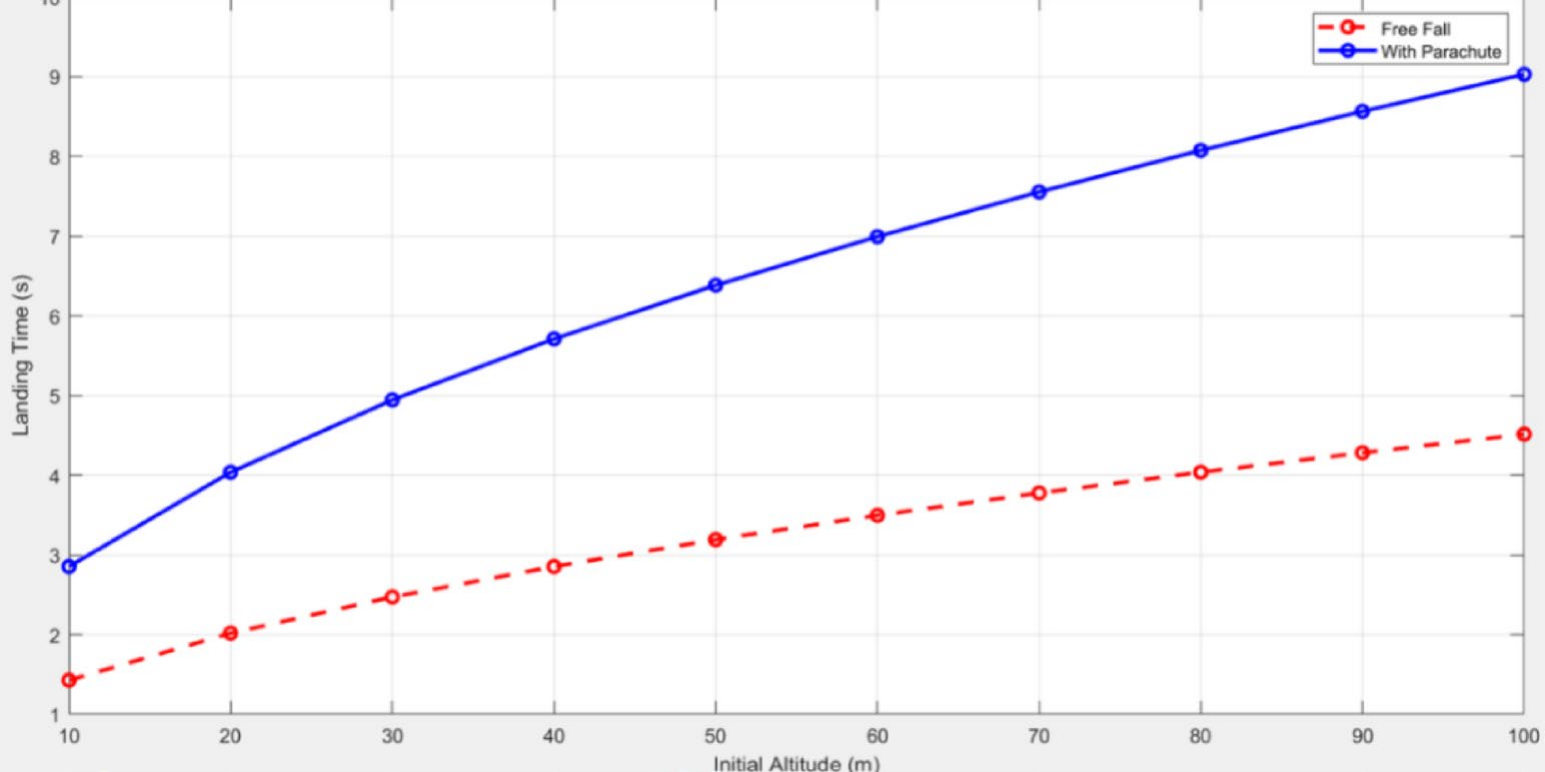
Descent time comparison from different altitudes, highlighting the effectiveness of the parachute in extending descent duration for safer landings.

Figure 11 compares the descent time of the UAV from varying altitudes in two scenarios—one without a parachute and another with the proposed parachute deployment system. In free fall, the descent time is determined using:

13 $$\:t=\sqrt{\frac{2h}{g}}$$

where *h* is the descent height and *g* is the gravitational acceleration. However, with a parachute, the drag force significantly alters the descent characteristics by increasing air resistance, which in turn increases the descent time. The figure shows that as the altitude increases, the difference in descent time between the two cases becomes more evident. The proposed system ensures that the UAV follows a slower, controlled descent, reducing impact forces and increasing recovery time.

**Fig. 12:**
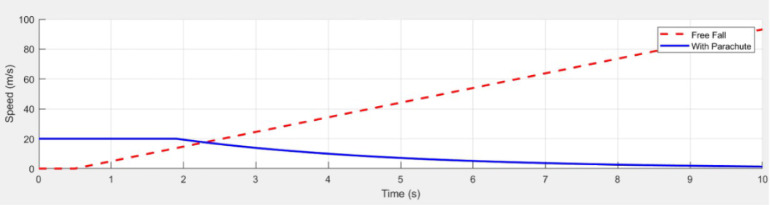
Descent speed comparison with and without a parachute.

**Fig. 13:**
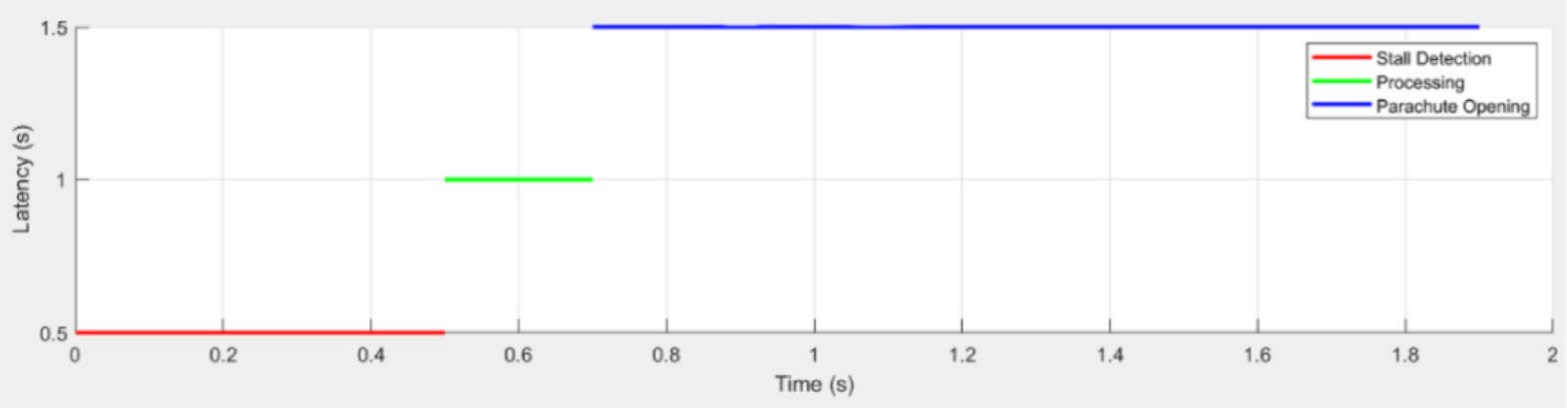
Parachute deployment latency at each stage.

Figure 12 consists graph—one showing speed comparison and Fig. 13 the illustrating the latency in parachute deployment. The speed comparison accounts for the effect of parachute opening latency, which causes the UAV to initially experience free-fall acceleration before the parachute inflates. During this phase, the UAV accelerates according to:

14 $$v\,=\,u+gt$$

The findings of this study have several significant implications for UAV safety, flight reliability, and future drone technology development. By integrating real-time stall detection and parachute-based emergency response mechanisms, UAVs can achieve higher levels of operational safety, reducing both economic and functional risks associated with crashes. The study demonstrates that parachute deployment at sufficient altitude (15 m) significantly reduces impact velocity, increasing the probability of drone survival. This has critical implications for commercial UAV applications such as package delivery, where drone integrity is vital for cost efficiency and service reliability. Industrial and inspection drones operating over hazardous areas, such as power lines or chemical plants, would also benefit from improved safety measures to prevent equipment loss. The stall detection and recovery system can be crucial for UAVs deployed in high-risk missions, such as military reconnaissance, disaster response, or autonomous search-and-rescue operations. Drones navigating unpredictable environments, such as mountainous terrain or post-disaster rubble, could benefit from rapid-response safety mechanisms that prevent critical failures. With increasing regulatory requirements for UAV operations, especially in urban and densely populated areas, integrating parachute-based safety systems can help meet aviation authority safety standards. By demonstrating controlled emergency descent capabilities, UAV operators may gain easier approval for flights in restricted zones, further enabling the expansion of commercial drone operations. By preventing severe crashes, the system reduces the frequency of costly drone repairs and replacements. This is particularly beneficial for large-scale drone operations, where maintenance costs directly impact profitability. In industrial applications such as agriculture, where fleets of drones monitor crops, minimizing downtime through improved crash resilience ensures consistent productivity. Pilots and autonomous flight planners can use stall detection data to optimize flight paths and prevent unnecessary stall-inducing maneuvers. This is particularly useful in racing drones, aerial cinematography, and high-speed UAV applications where aggressive flight patterns can lead to instability. While the study demonstrates the effectiveness of parachute deployment at higher altitudes, it also highlights critical challenges at lower altitudes, particularly below 10 m. At such heights, the parachute does not have sufficient time to fully deploy, resulting in high-impact crashes. Additionally, due to aerodynamic instabilities, the UAV may topple instead of descending uniformly, further diminishing the parachute’s effectiveness. This underscores the need for alternative emergency recovery methods, such as motor cut off, deployable airbags, or rapid rotor braking systems to mitigate crash damage. Future studies should explore hybrid safety mechanisms that combine multiple recovery strategies, ensuring effective emergency responses across all altitude ranges.

## Conclusion

This study successfully developed and validated a multi-parameter stall detection and emergency recovery system for UAVs, demonstrating substantial improvements in flight safety and survivability during critical failure scenarios. The proposed system integrates an STM32-based flight controller with gyroscope- and accelerometer-based stall detection, augmented by angular velocity, vertical acceleration, and motor saturation measurements to accurately identify unrecoverable stalls. The hybrid emergency response—parachute deployment for altitudes above 15 m and motor-based low-altitude impact mitigation mode below this threshold—ensures optimal survival outcomes across a wide range of operational conditions.

Simulation and Hardware-in-the-Loop (HIL) testing revealed that parachute-assisted recovery from 25 m reduced impact velocity from 22.2 m/s to 3.2 m/s, resulting in a 95% survival probability, whereas deployment at 10 m provided insufficient deceleration, yielding only 40% survivability. The stall detection algorithm achieved 100% accuracy across 200 test flights, with a rapid response latency of 0.08 s, highlighting the system’s reliability and deterministic behavior. These results underscore the critical importance of defining precise stall thresholds, as miscalibration can lead to either false positives or undetected stall events, jeopardizing UAV safety. In addition to confirming the effectiveness of altitude-aware parachute deployment, this study demonstrates the advantages of multi-parameter evaluation over conventional single-threshold methods, particularly under challenging conditions such as gusty winds, sensor noise, and aggressive maneuvers. The system’s ability to transmit GPS coordinates upon landing further enhances post-incident recovery and operational efficiency. For future work, the system could integrate hybrid low-altitude recovery mechanisms such as deployable airbags or rotor braking, and implement adaptive stall thresholds that adjust dynamically to payload, wind conditions, and flight mode. Overall, the proposed system represents a significant step toward enhancing UAV operational safety, mitigating economic and functional risks, and enabling reliable deployment in commercial, industrial, and high-risk applications. By combining rule-based stall detection, altitude-aware parachute deployment, and low-altitude motor cutoff, this study establishes a versatile framework for safer autonomous UAV operations.

## Data Availability

The datasets used and/or analysed during the current study are available from the corresponding author on reasonable request.
